# Interfacial Coupling and Reconstruction in Glycerolate-Derived Co–Mn Selenium-Treated Products for High-Performance Supercapacitors

**DOI:** 10.1021/acsomega.6c07974

**Published:** 2026-09-02

**Authors:** Tsai-Mu Cheng, Yu-Yan Ciou, Yan-Yu Liu, Chutima Kongvarhodom, Sibidou Yougbaré, Muhammad Saukani, Hung-Ming Chen, Lu-Yin Lin

**Affiliations:** † Graduate Institute for Translational Medicine, College of Medical Science and Technology, Taipei Medical University, Taipei 11031, Taiwan; ‡ Taipei Heart Institute, Taipei Medical University, Taipei 11031, Taiwan; § Cardiovascular Research Center, Taipei Medical University Hospital, Taipei 11031, Taiwan; ∥ Department of Chemical Engineering and Biotechnology, National Taipei University of Technology, Taipei 106344, Taiwan; ⊥ Department of Chemical Engineering, 65128 King Mongkut’s University of Technology Thonburi, 126 Pracha-u-thit, Toong-kru, Bangkok 10140, Thailand; # Institut de Recherche en Sciences de la Santé (IRSS-DRCO)/Nanoro, 03 B.P 7192, Ouagadougou 037192, Burkina Faso; ∇ Department of Mechanical Engineering, Faculty of Engineering, Universitas Islam Kalimantan MAB, Jl. Adhyaksa No. 2, Banjarmasin 70124, Kalimantan Selatan, Indonesia; ○ Gingen Technology Co., LTD., Rm. 7, 10F., No. 189, Sec. 2, Keelung Rd., Xinyi Dist., Taipei 11054, Taiwan

## Abstract

The development of electrode materials with efficient charge transfer and abundant interfacial active sites remains a key challenge for high-performance supercapacitors. Compared with conventional inorganic precursors, glycerolate-derived materials provide tunable coordination environments and self-templated structural evolution, enabling the formation of porous architectures with abundant active interfaces after post-synthetic conversion. A bimetallic cobalt–manganese glycerolate (CoMn-G) is utilized as a precursor to construct Co–Mn-based selenium-treated products via a solvothermal selenization process, with particular emphasis on interfacial reconstruction and electronic modulation. Incorporating Mn regulates the coordination environment, resulting in a homogeneous amorphous framework with enhanced defect features. Upon selenization, a pronounced phase transformation and morphological reconstruction occur, leading to the formation of porous architectures with enriched interfacial regions. Meanwhile, strong electronic coupling between Co and Mn induces charge redistribution, facilitating improved charge transfer kinetics. As a result, the optimized CoMn–Se electrode delivers a GCD-derived specific capacitance of 1015.8 F/g at 1 A/g, while a capacitance of 927.3 F/g is obtained from CV measurements at 10 mV/s along with a reduced charge transfer resistance. When assembled into a two-electrode device with reduced graphene oxide as the negative electrode, the system achieves an energy density of 66 Wh/kg at 725 W/kg and maintains 89% capacitance retention with 99% Coulombic efficiency after 10,000 cycles. The enhanced performance is attributed to synergistic effects of bimetallic interaction and selenization-induced interfacial evolution. This work provides insights into designing glycerolate-derived materials with tailored interfacial structures and electronic properties for advanced energy storage applications.

## Graphical abstract

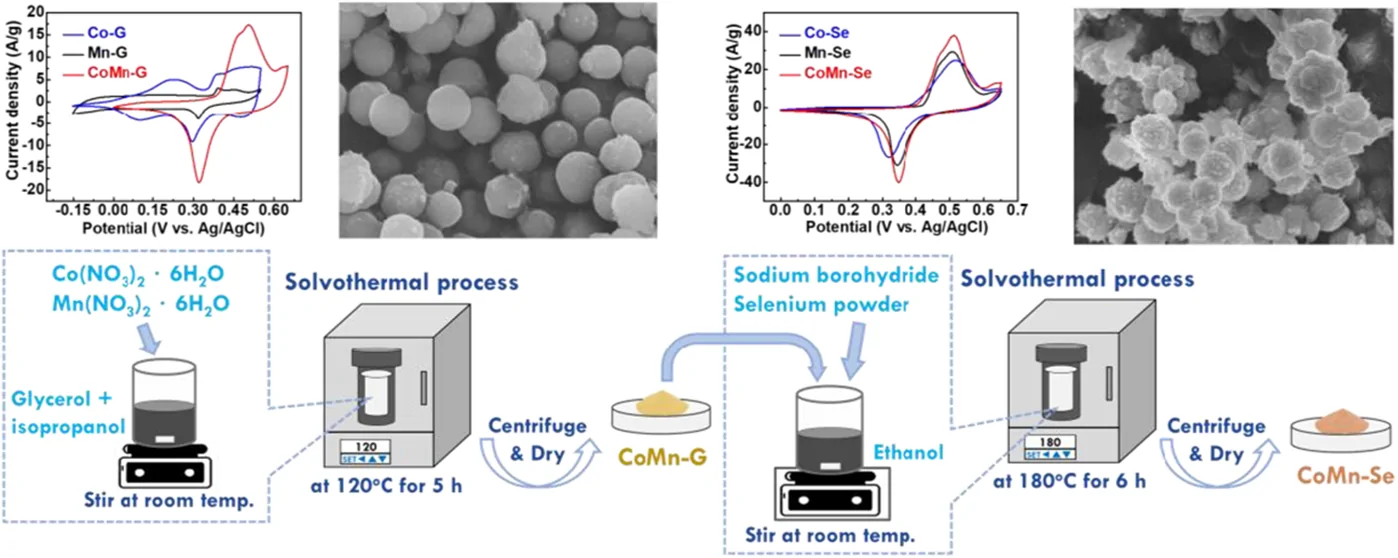

## 1 Introduction

The rapid development of modern electronics and renewable energy systems has significantly increased the demand for efficient and reliable energy storage technologies.^1^ Among various systems, batteries and supercapacitors represent two major categories with complementary characteristics. Batteries typically offer high energy density but suffer from limited power density and slower charge–discharge rates.^2–5^ In contrast, supercapacitors exhibit outstanding power density, fast charge–discharge capability, and excellent cycling stability, making them particularly attractive for applications requiring rapid energy delivery and long-term durability.^6–9^ Despite these advantages, the relatively low energy density of supercapacitors remains a key limitation. Since the electrochemical performance of supercapacitors is highly dependent on the properties of electrode materials, the rational design and development of advanced active materials play a decisive role in improving their overall performance.^10–13^

Recently, metal glycerolates and their derived materials have attracted increasing attention as promising electrode candidates for supercapacitors, owing to their tunable chemical composition, versatile coordination environments, and their ability to transform into functional nanostructures through post-synthetic treatments.^14–16^ In particular, glycerolate compounds can serve not only as active materials but also as structural templates for constructing complex architectures.^17,18^ Kubendhiran et al. utilized NiCo-glycerolate spheres as sacrificial templates to fabricate honeycomb-like CoNiMo layered double hydroxides, demonstrating the structural advantages of glycerolate-derived architectures for energy-related applications.^19^ Similarly, Kuo et al. systematically investigated cobalt and nickel glycerolates synthesized from fluorine-containing precursors, revealing that both the metal species and BF_4_^–^ anions play critical roles in modulating redox activity and charge storage behavior.^20,21^ These studies highlight that glycerolate-based systems provide a flexible platform for tailoring electrochemical properties through precursor design and structural control. Although these studies demonstrated the structural versatility of glycerolate-derived materials, they mainly focused on single-metal systems or morphology optimization, while systematic regulation of interfacial electronic structures through bimetallic design remains limited. Despite these advantages, the practical performance of metal glycerolates is still limited by their relatively low electrical conductivity and insufficient intrinsic electrochemical activity. To address these issues, incorporating transition metals with rich redox chemistry has been widely explored. Cobalt is known for its high theoretical capacitance and fast redox kinetics, while manganese offers multiple valence states, low cost, and environmental benignity, making it an attractive complementary component.^22–25^ Previous studies have demonstrated that modifying cobalt-based glycerolates, such as introducing fluorine species to form cobalt fluoride during synthesis, can effectively enhance electrochemical performance.^12^ In addition, glycerolate structures have been employed as precursors or templates for constructing metal selenides with advanced morphologies. Zardkhoshoui et al. reported copper–cobalt selenide hollow spheres derived from glycerate templates, showing improved electrochemical behavior due to their hollow structure and enhanced surface accessibility.^26^ Furthermore, Xu et al. synthesized cobalt diselenide (CoSe_2_) and manganese selenide (MnSe) nanosheets from CoMn-based precursors, demonstrating that bimetallic selenides can deliver superior electrochemical activity and abundant redox reactions compared to their single-metal counterparts.^27–30^ Although numerous Co–Mn-based selenide electrodes have been reported for supercapacitor applications, most studies focus on conventional binary metal selenides derived from hydroxides, oxides, or metal–organic framework precursors. In contrast, considerably less attention has been devoted to the phase evolution of glycerolate-derived Co–Mn precursors during selenization, particularly when the reaction results in mixed selenium oxide phases rather than conventional metal selenides. Since the crystal structure and anion chemistry strongly influence the electronic structure, redox behavior, and ion-transport characteristics of transition-metal compounds, understanding the electrochemical properties of these unconventional selenium oxide phases is of considerable interest. Therefore, this work focuses on correlating the formation of glycerolate-derived cobalt–manganese selenium oxide phases with their charge-storage performance.

Based on the above considerations, an important challenge remains to develop glycerolate-derived bimetallic selenides capable of simultaneously optimizing composition, interfacial electronic structure, and porous morphology to enhance electrochemical charge storage. In this work, a bimetallic cobalt–manganese glycerolate (CoMn-G) is utilized as a precursor for the synthesis of CoMn-Se via a solvothermal selenization process. The coexistence of Co and Mn within the glycerolate framework is expected to induce synergistic redox interactions and multiple valence states, thereby enhancing charge storage capability. Meanwhile, the glycerolate matrix acts as a self-template, facilitating the formation of structured architecture with abundant active sites. During selenization, in situ generated selenium species react with the metal centers, converting the precursor into conductive metal selenium-treated products with improved reaction kinetics. Compared to oxides or hydroxides, selenium-treated products generally exhibit higher electrical conductivity and more efficient ion transport. The structural transformation is also expected to retain or evolve porous features, promoting electrolyte accessibility and improving active material utilization. To clarify the respective roles of bimetallic synergy and selenization, monometallic glycerolates and their corresponding selenized counterparts are systematically investigated. In this context, the development of bimetallic Co–Mn glycerolate-derived selenium-treated products with tailored architectures represents a promising strategy for advancing high-performance supercapacitor electrodes. This study provides both scientific insight and practical guidance for designing next-generation electrode materials with enhanced electrochemical performance.

## 2 Experimental Section

### 2.1 Preparation of Cobalt Glycerolate, Manganese Glycerolate and Cobalt Manganese Glycerolate

Cobalt glycerolate (Co–G), manganese glycerolate (Mn–G), and bimetallic cobalt–manganese glycerolate (CoMn–G) were prepared through a solvothermal-assisted coordination process between metal ions and glycerol. In a typical synthesis of Co–G, 0.5 mmol of cobalt nitrate hexahydrate (Co­(NO_3_)_2_·6H_2_O, Acros, 98%) was introduced into a mixed solvent composed of 10 mL of glycerol (Echo, 99.5%) and 70 mL of isopropanol (Echo, 99.5%). The mixture was subjected to ultrasonic treatment for 10 min to ensure complete dissolution and formation of a homogeneous solution. The resulting solution was then sealed in a Teflon-lined reactor and maintained at 120 °C for 5 h to promote coordination and nucleation. After the system cooled naturally to ambient temperature, the resulting solid was isolated by centrifugation at 10,000 rpm for 2 min, followed by repeated rinsing with deionized water (DIW) and ethanol to remove residual species. The collected product was dried at 60 °C for 4 h. After washing and drying, an isolated yield of approximately 60.2% was obtained based on the total metal precursors. On the other hand, Mn–G was synthesized by an identical procedure, except that manganese nitrate hexahydrate (Mn­(NO_3_)_2_·6H_2_O, thermos scientific, 98%) was used to replace cobalt nitrate hexahydrate as the metal precursor. After washing and drying, an isolated yield of approximately 70.3% was obtained based on the total metal precursors. In the case of CoMn–G, 0.5 mmol of cobalt nitrate hexahydrate and 0.5 mmol of manganese nitrate hexahydrate were co-dissolved in the same solvent system, and the subsequent steps remained unchanged. After washing and drying, an isolated yield of approximately 65.1% was obtained based on the total metal precursors. The experimental processes for synthesizing Co-G, Mn-G, and CoMn-G were shown in Scheme 1.

**Scheme 1:**
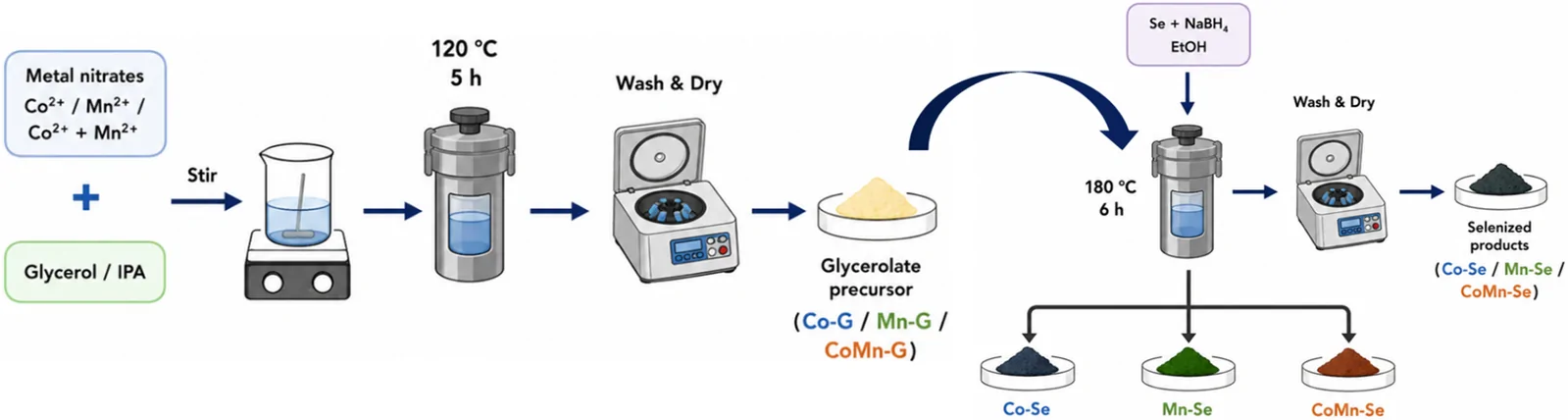
Schematic Illustration of the Preparation of Co-G, Mn-G, CoMn-G and their Corresponding Selenized Products (Co–Se, Mn–Se and CoMn-Se)

### 2.2 Preparation of Selenized Products

The selenized materials including selenized cobalt glycerolate (Co–Se), selenized manganese glycerolate (Mn–Se), and selenized cobalt–manganese glycerolate (CoMn–Se) were obtained via a solution-mediated reduction–selenization process using the corresponding glycerolate precursors. Briefly, 100 mg of the as-prepared glycerolate precursor (Co–G, Mn–G or CoMn–G) was dispersed in 30 mL of ethanol containing 80 mg of selenium powder (Se, 99.5%, Alfa Aesar) and 106 mg of sodium borohydride (98%, Thermoscientific). The suspension was magnetically stirred for 1 h to facilitate precursor dispersion and in situ generation of reactive selenium species. The mixture was then transferred to a sealed autoclave and heated at 180 °C for 6 h, allowing the selenization reaction to proceed under solvothermal conditions. Upon completion, the solid products were retrieved by centrifugation and thoroughly washed with ethanol multiple times to eliminate unreacted residues. The obtained powders were then dried and used for subsequent characterization. The experimental processes for synthesizing Co–Se, Mn–Se, and CoMn-Se were shown in Scheme 1.

### 2.3 Electrode Fabrication and Assembly of Energy Storage Device

The energy storage electrodes were prepared by depositing a homogeneous slurry onto nickel foam as the current collector. Prior to use, the nickel foams were pre-cleaned to remove surface impurities. The slurry was formulated by mixing the active material, conductive carbon black, and poly­(tetrafluoroethylene) (PTFE, Polysciences, Inc.) binder in a mass ratio of 7:2:1. A total of 70 mg of solid components was dispersed in 1.5 mL of a mixed solvent containing ethanol and DIW (1:1, v/v), followed by continuous stirring at ambient temperature for 24 h to obtain a uniform suspension.^31^ The resulting mixture was evenly spread onto the nickel foam and subsequently dried at 60 °C overnight to ensure adequate adhesion and solvent removal. The rGO negative electrode was prepared using the same slurry-coating procedure as that employed for the positive electrode. Briefly, rGO powder, acetylene black, and PVDF were mixed in a weight ratio of 8:1:1 using *N*-methyl-2-pyrrolidone (NMP) as the solvent. The resulting slurry was coated onto nickel foam, dried at 80 °C overnight, and pressed before electrochemical testing. The active-material loading was approximately 0.5 mg/cm^2^. Prior to assembling the asymmetric supercapacitor, the electrochemical performance of the rGO electrode was evaluated in a three-electrode configuration. The specific capacitance of around 90.3 F/g was obtained at 10 mV/s. The mass ratio between the positive CoMn-Se electrode and the negative rGO electrode was calculated according to eq 1, where the specific capacitances and potential windows of the individual electrodes were used to satisfy the charge-balance relationship. For device assembly, the optimized electrode was employed as the positive electrode, while a reduced graphene oxide (rGO) electrode served as the negative counterpart. An aqueous 3 M KOH solution was used as the electrolyte. The charges stored between two electrodes are balance due to the charge balance principle (*q* ^+^ = *q* ^–^), where the mass loading of the positive and negative electrodes is adjusted according to their specific capacitance and operating potential window to ensure balanced charge storage in the assembled asymmetric device. Therefore, the mass ratio of active materials on both electrodes was determined according to eq 1 as follows.^32^

1 $$\frac{m_{+}}{m_{-}}=\frac{C_{-}\cdot \Delta V_{-}}{C_{+}\cdot \Delta V_{+}}$$

where *C* represents the specific capacitance (F g^–1^), Δ*V* is the operating potential window (V), and *m* corresponds to the mass loading of the electrode (g).

### 2.4 Material and Electrochemical Characterizations

Structural and surface characteristics of the prepared samples were analyzed using multiple techniques. Field-emission scanning electron microscopy (FE-SEM, Nova NanoSEM 230, FEI, USA) was employed to observe morphology. The average particle diameter was determined by measuring approximately 100 randomly selected particles from the SEM images using ImageJ software. The phase identification was carried out by X-ray diffraction (XRD, X′Pert3 Powder, PANalytical). Surface chemical states and elemental composition were further investigated using X-ray photoelectron spectroscopy (XPS, VG Scientific ESCALAB 250) with an Al Kα radiation source. Electrochemical performance was evaluated in a conventional three-electrode system, using a platinum foil as the counter electrode and an Ag/AgCl electrode as the reference. Measurements including cyclic voltammetry (CV), galvanostatic charge–discharge (GCD), and electrochemical impedance spectroscopy (EIS) were conducted on an Autolab PGSTAT204 workstation equipped with an FRA2 module. Impedance spectra were recorded over a frequency range from 0.01 Hz to 100 kHz under open-circuit conditions. The specific capacitance (*C*_F_) and specific capacity were derived from the GCD profiles using the discharge time by eqs 2 and 3 as follows.^33^

2 $$C_{F}=\frac{I\times \Delta t}{\Delta V}$$

3 $$s\mathrm{pecific capacity}=C_{F}\times \Delta V=I\times \Delta t$$

where *I* is the applied current density (A g^–1^) and Δ*t* denotes the discharge duration (s). In addition, the specific capacitance based on CV measurements was determined by integrating the entire cyclic voltammogram (including both anodic and cathodic branches) according eq 4 as follows.^34,35^

4 $$C_{F}=\frac{\int \mathrm{IdV}}{2\Delta V\cdot \nu }$$

where ∫IdV represents the integrated area under the CV curve, and *ν* is the scan rate (V/s). It is noticed that Δ*V* is the actual operating potential window employed for the corresponding electrode during electrochemical testing. The Δ*V* value used for each sample corresponds to the potential window shown in the respective CV and GCD measurements rather than a fixed value. All electrochemical measurements were performed using at least three independently prepared electrodes (*n* ≥ 3). The reported values are presented as mean ± standard deviation (SD).

## 3 Results and Discussion

### 3.1 Material Analysis of Co-G, Mn-G, CoMn-G, Co–Se, Mn–Se, and CoMn-Se

Crystalline structure analysis is essential for verifying the phase composition of the active materials. Figure 1a presents the XRD patterns of Co-G, Mn-G, and CoMn-G, along with the reference patterns of Mn_3_O_4_ (JCPDS #01-080-0382),^36^ (Co,Mn)­(Co,Mn)_2_O_4_ (JCPDS #00-018-0410) and CoC_2_O_4_·2H_2_O (JCPDS #00-001-0296)^37^ for comparison. Co-G displays predominantly broad and featureless diffraction patterns, confirming its amorphous or poorly crystalline nature. This characteristic is commonly observed in metal-glycerate frameworks formed through coordination between metal ions and polyol ligands, where long-range periodicity is largely suppressed. In contrast, distinct diffraction peaks corresponding to the Mn_3_O_4_ phase can be clearly identified in Mn-G. The diffraction peaks appear at 2θ values of 18.0°, 29.0°, 31.1°, 32.4°, 36.2°, 38.2°, 44.6°, 50.9°, 54.0°, 55.5°, 58.6°, 59.9°, 64.7° and 74.4°, corresponding to the (101), (112), (200), (103), (211), (004), (220), (105), (312), (303), (321), (224), (314) and (413) crystal planes of Mn_3_O_4_, respectively. This phenomenon suggests partial oxidation and the formation of locally ordered crystalline domains during the synthesis process. Interestingly, CoMn-G does not exhibit any obvious diffraction peaks and instead shows a largely amorphous pattern similar to that of Co-G. This indicates that the incorporation of Co species effectively suppresses the crystallization of manganese oxide phases, leading to a more homogeneous and disordered coordination structure. These observations suggest that the incorporation of Co suppresses the crystallization of manganese oxide phases, resulting in a more homogeneous precursor structure. However, the actual elemental distribution cannot be directly confirmed from XRD alone and would require elemental mapping for verification. Such structural disorder is beneficial for providing abundant unsaturated coordination sites and is expected to facilitate subsequent selenization-induced reconstruction. After the selenization process, significant structural evolution is observed. Unlike most reported Co–Mn systems that predominantly yield binary metal selenides after selenization, the present synthesis results in the formation of mixed selenium oxide phases. This distinct phase evolution is likely associated with the oxidation state of selenium under the present solvothermal conditions and subsequent thermal treatment. The coexistence of multiple selenium oxide phases may alter the local coordination environment and electronic structure of the transition-metal centers, thereby influencing the electrochemical behavior. This feature distinguishes the present material from previously reported CoMn selenide electrodes and represents one of the key characteristics of this work.

**Figure 1:**
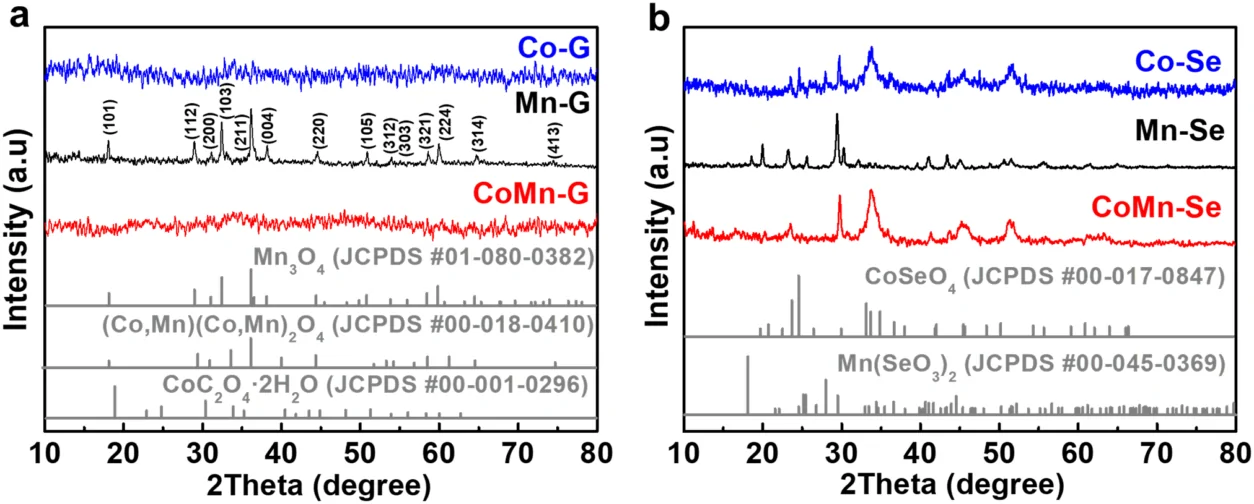
XRD patterns of (a) Co-G, Mn-G and CoMn-G and (b) Co–Se, Mn–Se and CoMn-Se.

Figure 1b shows the XRD patterns of Co–Se, Mn–Se and CoMn-Se, and the reference patterns of CoSeO_4_ (JCPDS #00-017-0847) and Mn­(SeO_3_)_2_ (JCPDS #00-045-0369) are also presented for comparison. The diffraction patterns of Co–Se, Mn–Se and CoMn-Se exhibit well-defined peaks with markedly enhanced intensity, indicating the formation of crystalline selenium-containing phases. The Co–Se can be indexed to CoSeO_4_ and Mn–Se corresponds to Mn­(SeO_3_)_2_, suggesting that selenium incorporation is accompanied by partial oxidation under solvothermal conditions. The formation of CoSeO_4_ and Mn­(SeO_3_)_2_ instead of pure metal selenides suggests that selenium undergoes a complex redox process during solvothermal treatment. Although NaBH_4_ acts as a reducing agent to convert elemental Se into reactive selenide species (Se^2–^), the reaction environment is not strictly inert. The presence of residual dissolved oxygen, nitrate ions from the metal precursors, and possible oxidative species generated during the reaction can lead to partial re-oxidation of Se^2–^ into oxyanions such as SeO_3_^2–^ or SeO_4_^2–^. Meanwhile, the metal ions (Co^2+^ and Mn^2+^) can also undergo oxidation under hydrothermal conditions, favoring the formation of metal-selenite or selenate compounds rather than pure metal selenides. Therefore, the coexistence of reduction and oxidation processes results in the formation of oxidized selenium-containing phases. The selenide phases obtained through related precursor-conversion or direct-synthesis routes have also been previously identified and clearly distinguished from oxidized Se–O compounds.^38–40^ Compared to elemental selenium or pure metal selenides, selenium-containing oxyanions (such as SeO_3_^2–^ or SeO_4_^2–^) can provide additional advantages for electrochemical energy storage.^41^ The presence of oxygen atoms introduces stronger metal–oxygen bonding and higher polarity, which enhances the interaction between the electrode surface and electrolyte ions, thereby improving wettability and facilitating ion diffusion. In addition, oxygen incorporation can modulate the electronic structure of selenium-based compounds, leading to more favorable charge redistribution and improved redox activity. The coexistence of metal-Se and metal-O bonds creates a hybrid electronic environment that can promote reversible redox reactions and stabilize intermediate species during cycling. Furthermore, oxygen-containing selenium species often exhibit improved structural stability compared to pure selenides, mitigating volume variation and structural degradation during repeated charge–discharge processes. Such stabilization is beneficial for long-term cycling performance. It is also worth noting that in many selenide-based systems, selenium itself does not directly participate in the pseudocapacitive reaction but plays a role in enhancing conductivity and facilitating phase transformation into electrochemically active oxyhydroxide species.^42^ Therefore, the presence of selenium–oxygen species may further promote the formation of active redox phases, leading to improved electrochemical performance. Notably, the diffraction peaks of CoMn-Se are largely consistent with those of Co–Se in both peak position and intensity, indicating that the crystalline structure is predominantly governed by the cobalt-based phase. No significant peak broadening or shift is observed, suggesting that the incorporation of Mn does not induce substantial lattice distortion. However, a characteristic diffraction feature around 30° can be clearly matched with the Mn–Se phase, confirming the successful incorporation of Mn species into the system. This result implies that Mn is introduced without altering the primary crystal framework of Co–Se, suggesting that Mn is incorporated without causing significant lattice distortion. However, the exact incorporation configuration cannot be conclusively determined from the present XRD results alone. In addition, no characteristic peaks of elemental selenium are detected, confirming the effective consumption of Se precursors. The transformation from amorphous glycerate to crystalline selenized phases suggests a reconstruction process involving the breakdown of the coordination framework and subsequent reorganization into thermodynamically stable structures.

The morphology and microstructural evolution of the as-prepared samples before and after selenization were investigated by SEM, as shown in Figure 2. Figure 2a–c shows the SEM images of Co-G, Mn-G and CoMn-G, respectively. Co-G exhibits a uniform distribution of spherical particles with an average diameter of approximately 1 μm. The surface of these spheres is decorated with vertically aligned nanosheets, forming a hierarchical structure that is characteristic of glycerate-derived materials formed via coordination-driven growth. This morphology suggests a relatively homogeneous nucleation and growth process governed by the coordination between Co^2+^ ions and glycerol molecules. In contrast, Mn-G displays a more irregular and aggregated morphology with partially collapsed structures, and a significantly larger particle size of around 3 μm. This difference can be attributed to the distinct chemical behavior of Mn species, which tend to undergo partial oxidation during synthesis, leading to the formation of Mn_3_O_4_ as confirmed by XRD. The presence of crystalline oxide domains likely accelerates anisotropic growth and particle coalescence, resulting in larger and less uniform structures. For the bimetallic CoMn-G, a relatively regular spherical morphology is observed with an average diameter of approximately 1.5 μm, which lies between those of Co-G and Mn-G. Notably, the surface appears smoother compared to Co-G, without the presence of obvious nanosheet substructures. The smoother surface morphology of CoMn-G can be attributed to the simultaneous coordination of Co^2+^ and Mn^2+^ with glycerol, which modifies the nucleation and growth kinetics during the solvothermal process. Compared with Co-G, the presence of Mn suppresses the anisotropic growth responsible for the vertically aligned nanosheet substructures. Meanwhile, compared with Mn-G, the incorporation of Co inhibits excessive crystallization and particle aggregation. As a result, the bimetallic coordination environment promotes a more isotropic growth process, leading to relatively uniform spherical particles with smoother surfaces. Meanwhile, the incorporation of Co inhibits the crystallization of Mn oxide species, as evidenced by the absence of distinct diffraction peaks in the XRD pattern. This synergistic effect leads to a more homogeneous and isotropic growth process in CoMn-G, resulting in intermediate particle size and a smoother surface morphology. The discrepancy in crystallinity among the three samples, where Co-G and CoMn-G remain largely amorphous while Mn-G exhibits oxide-related diffraction peaks, highlights the critical role of metal composition in regulating both structural ordering and morphological evolution.

**Figure 2:**
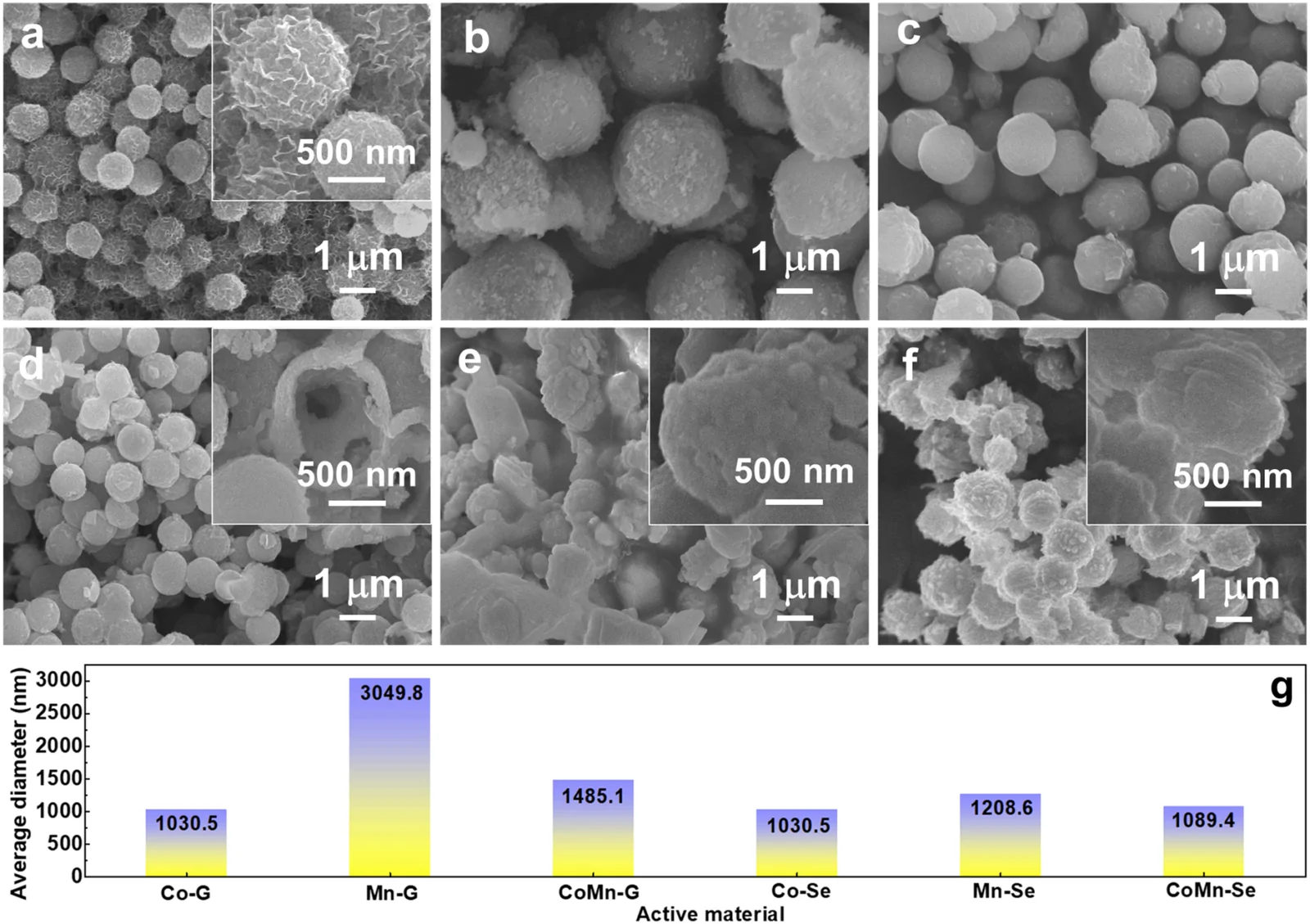
SEM images of (a) Co-G, (b) Mn-G, (c) CoMn-G, (d) Co–Se, (e) Mn–Se and (f) CoMn-Se; (g) relationship of average diameter and the active materials.

On the other hand, Figure 2d–f present the SEM images of Co–Se, Mn–Se and CoMn-Se, respectively. The enlarged images are also inserted in the corresponding figures. Pronounced morphological reconstruction was observed after the selenization process, indicating a dynamic structural evolution governed by interfacial diffusion and phase transformation. For Co–Se, the overall particle diameter remains comparable to that of Co-G. However, the vertically aligned nanosheet architecture disappears after selenization, and a distinct hollow interior is formed. The observed morphological evolution suggests diffusion-assisted interfacial reconstruction during the selenization process. Although the particle morphology appears consistent with structural evolution reported for Kirkendall-type diffusion in related systems, confirmation of an actual hollow interior would require TEM characterization. Therefore, the present interpretation should be regarded as a plausible mechanism rather than direct experimental evidence. Meanwhile, the collapse of the nanosheet framework suggests interfacial reconstruction driven by surface energy minimization during the conversion from glycerolate to selenium-treated product phases. Such hollow structures are expected to provide increased interfacial area and shortened ion transport pathways. In contrast, Mn–Se exhibits a highly aggregated and compact morphology composed of irregular particles. Compared to Mn-G, the particle size of Mn–Se decreases to approximately 1 μm, indicating structural densification accompanied by fragmentation. This behavior can be explained by the rapid nucleation and growth of Mn­(SeO_3_)_2_ crystalline domains during selenization, leading to particle coalescence and reduced structural integrity. The relatively high crystallinity as evidenced by its sharp XRD reflections suggests that Mn species preferentially reorganize into thermodynamically stable bulk phases, thereby suppressing the formation of porous or hierarchical structures. Notably, CoMn-Se shows a reduced particle size like that of Co–Se, along with a rough surface with interconnected nanostructures is observed by SEM, suggesting the formation of an open morphology that may facilitate electrolyte penetration. Nevertheless, quantitative evaluation of pore characteristics would require additional BET analysis, which is beyond the scope of the present study. This hierarchical morphology is indicative of a kinetically controlled growth process arising from the coexistence of Co and Mn species. The introduction of Mn is believed to modify the interfacial reaction kinetics and disrupt continuous crystal growth of the Co-based phase, thereby inhibiting excessive grain coarsening. Simultaneously, the competitive nucleation between Co- and Mn-containing phases generates structural heterogeneity, leading to the formation of abundant pores and surface roughness. Such features are characteristic of systems with strong interfacial interactions and multiphase evolution. These morphological characteristics are in good agreement with the XRD results. The diffraction peaks of CoMn-Se are largely consistent with those of Co–Se in both position and intensity, indicating that the crystalline structure is predominantly governed by the CoSeO_4_ phase. The absence of noticeable peak shifts or broadening suggests that Mn incorporation does not significantly alter the Co-based lattice, implying limited solid-solution formation and the presence of phase-separated domains. Meanwhile, the emergence of a characteristic peak at around 30° corresponding to Mn­(SeO_3_)_2_ confirms the successful incorporation of Mn species as a secondary phase. This phase heterogeneity is consistent with the observed porous and interconnected morphology, further supporting that interfacial interactions between distinct phases play a critical role in directing the structural evolution.

Figure 2g further shows the relationship of the average diameter and active materials. The distinct size evolution behaviors are observed following the selenization process. The average diameter of around 1030.5 nm for Co–Se remains nearly unchanged compared to that for Co-G, indicating that the overall particle framework is well preserved despite the internal structural reconstruction after the selenization process. This phenomenon implies that the conversion process is primarily governed by interfacial diffusion and topotactic transformation, rather than complete structural collapse. In contrast, Mn–Se exhibits a pronounced reduction in particle size of around 1208.6 nm, compared to its precursor Mn-G with the particle size of around 3049.8 nm. This phenomenon can be attributed to structural densification and fragmentation associated with the formation of crystalline Mn­(SeO_3_)_2_. The transition toward a thermodynamically stable crystalline phase likely induces volume contraction and particle breakage, consistent with the aggregated morphology observed. For the bimetallic system, CoMn-Se shows an average diameter of around 1089.4 nm, which is comparable to that of Co–Se with the average diameter of around 1030.5 nm. This result suggests that the Co-based component dominates the preservation of the macroscopic particle framework. Nevertheless, a markedly rougher and more porous surface is observed for CoMn-Se, indicating that Mn incorporation plays a critical role in modulating the interfacial reaction kinetics and microstructural evolution. The coexistence of Co and Mn species likely induces competitive nucleation and heterogeneous phase formation during selenization, which suppresses excessive grain growth while generating abundant nanoscale features. This interpretation is consistent with the XRD results, where CoMn-Se is primarily governed by the Co-based crystalline phase, while Mn exists as a secondary phase without inducing significant lattice distortion. Such a phase distribution indicates that structural evolution is governed by interfacial interactions between distinct domains instead of homogeneous lattice incorporation, resulting in a size-retained yet structurally complex and porous architecture.

To examine the surface composition and interfacial electronic structure evolution of the glycerate precursors before and after selenization, XPS spectra was then measured and analyzed, as shown in Figure 3. The survey spectra of Co-G, Mn-G, and CoMn-G are respectively shown in Figure 3a–c, confirming the presence of Co, O, and C in Co–G, Mn, O, and C in Mn–G, and the coexistence of Co, Mn, O, and C in CoMn–G. Figure 3d–f shows the survey spectra of Co–Se, Mn–Se, and CoMn-Se, respectively. In addition to the elements observed in their corresponding precursors, a distinct Se signal is detected in all samples, confirming the successful formation of metal selenium-treated products. Furthermore, the high-resolution Co 2p spectra of Co-G and CoMn-G (Figure 3g,h) exhibit the characteristic Co 2p_3/2_ and Co 2p_1/2_ doublets together with shake-up satellite features, confirming the coexistence of Co^2+^/Co^3+^ species. No obvious binding-energy shift is observed between Co-G and CoMn-G, suggesting that the local chemical environment of Co remains largely unchanged after Mn incorporation. The binding energy of Co^2+^ in Co 2p_3/2_ shifts from 780.8 V for Co-G to 780.6 eV for CoMn-G. Also, the binding energy of Co^3+^ in Co 2p_3/2_ shifts from 779.2 V for Co-G to 779.1 eV for CoMn-G. The slight shifts in both Co oxidation states imply changes in the local electronic structure of Co induced by Mn incorporation, reflecting an electronic interaction between the two metal centers. In contrast, the Mn 2p spectra (Figure 3i,j) show a noticeable positive shift of the Mn^4+^ 2p_3/2_ component from 642.8 eV for Mn-G to 644.1 eV for CoMn-G. This shift suggests that the electronic environment surrounding the Mn species is modified after the introduction of Co, indicating an interaction between the two metal species. However, since no corresponding shift is observed for the Co 2p spectra, the present XPS results provide only qualitative evidence of changes in the Mn electronic environment rather than definitive proof of strong electronic coupling. Figure S1­(a)–(d) in the Supporting Information presents the corresponding Co 2p and Mn 2p spectra of the selenized samples. Notably, the Co 2p and Mn 2p signals of the selenized samples are extremely weak or nearly undetectable (Figure S1), indicating that the corresponding metal species are not readily detected within the XPS probing depth. However, the simultaneously weak Se 3d signals observed for Co–Se and CoMn-Se suggest that the attenuation of the metal signals cannot be attributed solely to the formation of a selenium-rich outer layer. Instead, these observations are more consistent with a heterogeneous near-surface composition, where oxygen-containing species and adventitious carbon formed during post-synthesis handling or air exposure partially cover the outermost surface, thereby attenuating both the metal and selenium photoelectron signals. Since depth-profiling XPS or elemental mapping was not performed in this study, the detailed elemental distribution within the surface region cannot be conclusively determined. Therefore, the present XPS results are interpreted only as evidence of surface reconstruction following selenization rather than as proof of a selenium-enriched outer layer. The O 1s spectra (Figure 3k–p) can be deconvoluted into three components, including lattice oxygen, defective oxygen, and adsorbed oxygen. Compared with single-metal samples, CoMn-G exhibits an increased fraction of defective oxygen, indicating that bimetallic coordination induces local structural distortion and generates more surface-active sites, which is critical for interfacial reactions. After selenization, the O 1s spectra of Co–Se show an almost complete disappearance of lattice oxygen signals, whereas Mn–Se and CoMn-Se exhibit additional components assignable to Se–O bonding. Meanwhile, a reduced contribution from lattice oxygen and an increased proportion of higher binding energy components are observed, indicating partial substitution of oxygen by selenium and the generation of defect-rich surfaces. The presence of Se–O species in Mn-containing samples suggests that selenium is more prone to surface oxidation or forms oxy-selenide species when interacting with Mn, compared to Co-only systems. The oxidized SeO_3_^2–^ and SeO_4_^2–^ species are attributed to the partial surface oxidation of selenium during or after the solvothermal process. Although NaBH_4_ acts as a strong reducing agent, complete reduction of all selenium species is not guaranteed because residual dissolved oxygen and nitrate ions from the metal nitrate precursors may compete with the reduction process. Furthermore, slight surface oxidation may occur during the washing, drying, and air exposure steps. Since XPS is a surface-sensitive technique, these oxidized selenium species mainly represent surface oxidation rather than the bulk composition. This difference may arise from the distinct metal–oxygen bond strengths and redox properties of Mn and Co, leading to varied surface reconstruction behaviors during selenization. Overall, these results reflect a significant reorganization of the local coordination environment induced by the reduction-selenization process. Lastly, the Se 3d spectra of Co–Se, Mn–Se, and CoMn-Se are respectively shown in Figure 3q–s. The Mn–Se exhibits characteristic doublets at 52 to 55 eV corresponding to Se 3d_5/2_ and Se 3d_3/2_, confirming the formation of metal–selenium bonding. In contrast, the Se spectra of Co–Se and CoMn-Se present negligible signals associated with Se 3d, suggesting that selenium species are scarcely exposed within the XPS probing depth (around 5 to 10 nm). The weak Se 3d intensity observed for Co–Se and CoMn-Se indicates that selenium-containing species are not strongly represented within the outermost XPS sampling region. Combined with the attenuated Co and Mn signals, this observation suggests that the near-surface region is compositionally heterogeneous and likely contains oxygen-rich surface species together with adventitious carbon accumulated after synthesis and air exposure. Because no depth-profiling XPS or elemental mapping measurements were conducted, the precise surface distribution of selenium cannot be determined, and the present interpretation is therefore limited to qualitative evidence of surface reconstruction after selenization. Overall, the XPS results reveal that bimetallic incorporation induces significant electronic interaction between Co and Mn, leading to charge redistribution and defect formation, while the subsequent selenization further tailors the surface chemistry and coordination environment. These features are expected to modulate interfacial charge transfer behavior and enhance catalytic activity.

**Figure 3:**
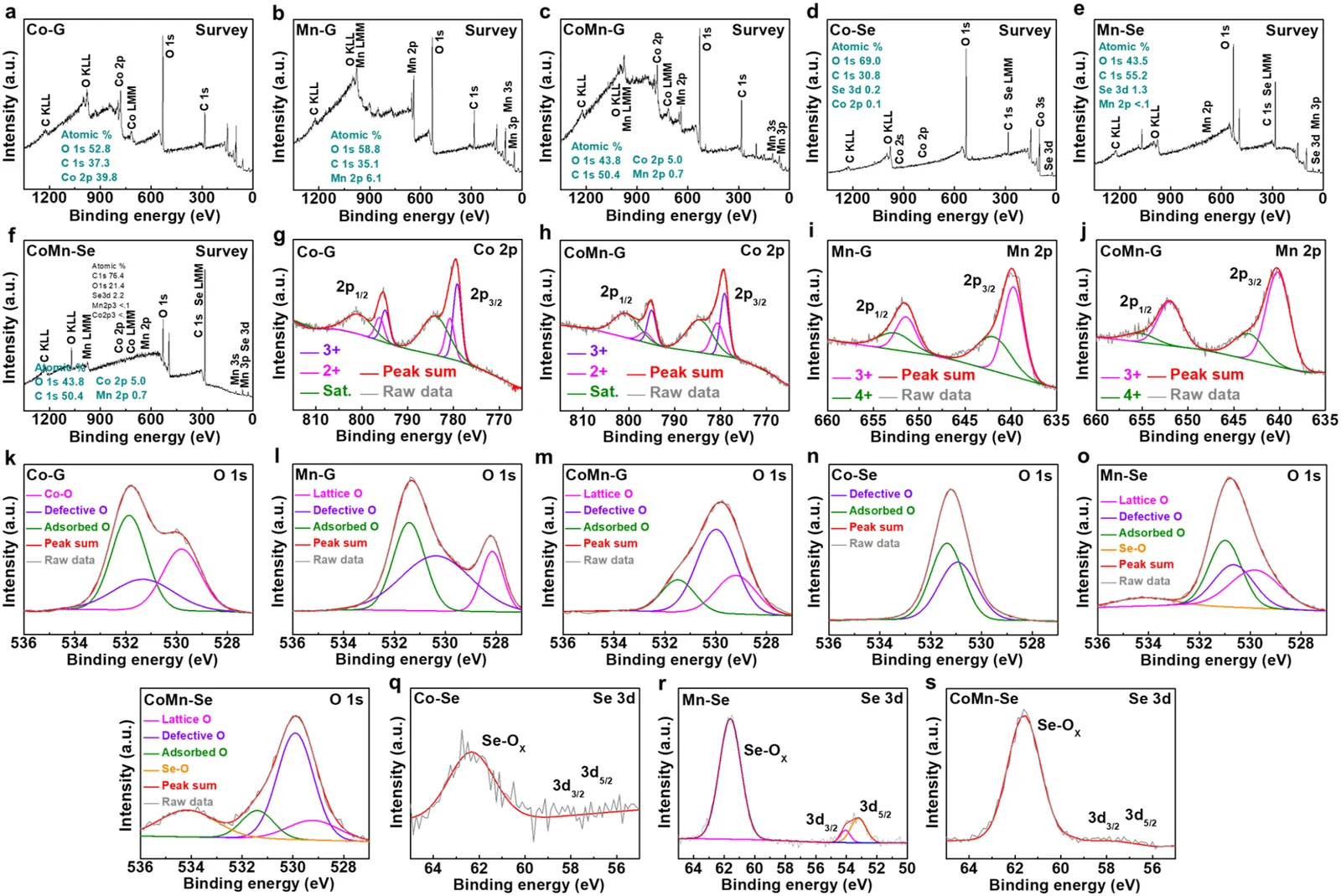
XPS spectra of Co-G, Mn-G, CoMn-G, Co–Se, Mn–Se and CoMn-Se: (a–f) survey spectra; (g, h) Co 2p; (i, j) Mn 2p; (k–p) O 1s; (q–s) Se 3d.

### 3.2 Electrochemical Analysis of Co-G, Mn-G, CoMn-G, Co–Se, Mn–Se, and CoMn-Se

The electrochemical behaviors of the Co-G, Mn-G, and CoMn-G electrodes were first investigated by using cyclic voltammetry, as shown in Figure 4a. It should be noted that CoMn-G exhibits a different suitable potential window compared to Co-G and Mn-G. Therefore, a potential range of 0.00 to 0.65 V vs. Ag/AgCl was specifically adopted for CoMn-G. All three electrodes exhibit pronounced redox peaks rather than ideal rectangular shapes, indicating that the charge storage mechanism is dominated by Faradaic pseudocapacitive reactions. The observed redox peaks are associated with reversible oxidation-reduction transitions of Co and Mn species in alkaline electrolyte. For Co-G, the reactions can be described as Co­(OH)_2_ + OH^–^ ↔ CoOOH + H_2_O + e^–^ and CoOOH + OH^–^ ↔ CoO_2_ + H_2_O + e^–^. For Mn-G, the reactions are Mn­(OH)_2_ + OH^–^ ↔ MnOOH + H_2_O + e^–^ and MnOOH + OH^–^ ↔ MnO_2_ + H_2_O + e^–^. A comparison of the CV curves reveals that Mn-G exhibits a relatively weaker current response and smaller enclosed area than Co-G, indicating inferior electrochemical activity of Mn-G. In contrast, CoMn-G exhibits the highest current response and largest integrated area, indicating significantly enhanced charge storage capability arising from the synergistic interaction between cobalt and manganese species. Quantitatively, the *C*_F_ values calculated from CV curves (Table 1) are 462.2, 203.0, and 570.2 F/g for Co-G, Mn-G, and CoMn-G, respectively, with corresponding specific capacities of 89.9, 39.0, and 103.3 mAh/g. These results indicate that Mn-G suffers from limited electrochemical activity, whereas the incorporation of Mn into Co-based materials significantly enhances capacitance through synergistic redox interactions.

**Figure 4:**
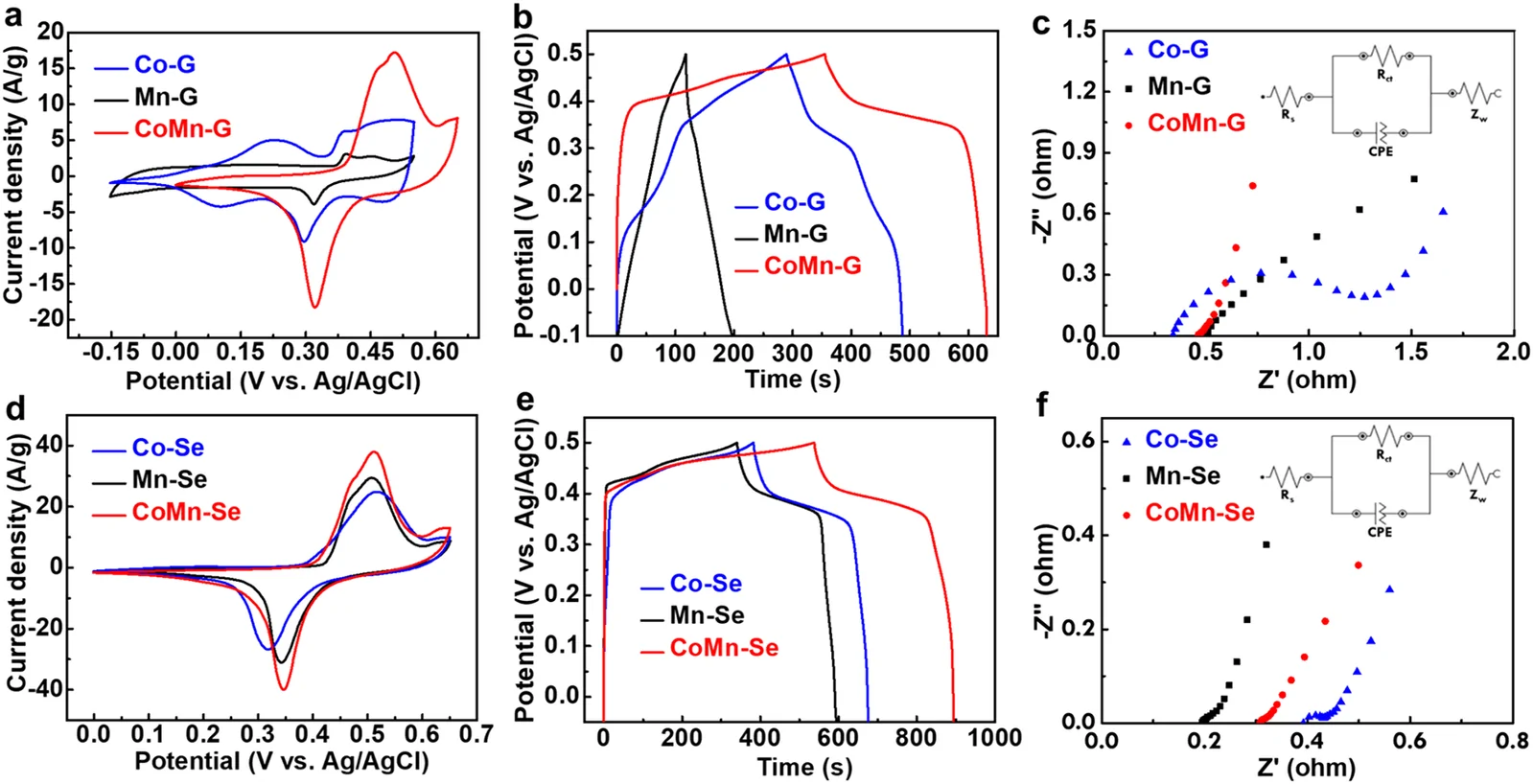
Electrochemical performance of glycerolate and selenized electrodes: (a, d) CV curves, (b, e) GCD curves, and (c, f) Nyquist plots with the corresponding equivalent circuits for Co-G, Mn-G, CoMn-G and Co–Se, Mn–Se, CoMn-Se, respectively.

**Table 1:** Electrochemical Parameters of Co-G, Mn-G, CoMn-G, Co–Se, Mn–Se, and CoMn-Se Electrodes^a^

active material	*C*_F_ (F/g) (CV)	capacity (mAh/g) (CV)	*C*_F_ (F/g) (GCD)	capacity (mAh/g) (GCD)	*R*_s_ (Ω)	*R*_ct_ (Ω)	loading mass (mg)
Co-G	462.2 ± 15.7	89.9 ± 3.0	430.7 ± 14.5	71.8 ± 2.4	0.34	0.90	5.2
Mn-G	203.0 ± 7.5	39.0 ± 1.3	170.2 ± 8.3	28.4 ± 1.4	0.51	1.62	4.8
CoMn-G	570.2 ± 15.0	103.0 ± 3.2	606.2 ± 14.0	101.0 ± 2.3	0.47	0.08	5.0
Co–Se	777.5 ± 20.1	140.4 ± 3.8	840.1 ± 25.1	128.3 ± 3.8	0.30	0.05	5.1
Mn–Se	714.5 ± 19.5	129.0 ± 4.0	715.0 ± 24.3	109.2 ± 3.7	0.17	0.07	4.9
CoMn-Se	927.3 ± 20.2	167.4 ± 3.4	1015.8 ± 31.9	155.2 ± 4.9	0.28	0.02	4.8

a CV curves are measured at 10 mV/s. GCD curves are measured at 1 A/g.

The GCD curves of the Co-G, Mn-G, and CoMn-G electrodes shown in Figure 4b also confirm their pseudocapacitive behaviors. All electrodes exhibit distinct potential plateaus corresponding to Faradaic reactions, which are consistent with the redox peaks observed in the CV curves. This phenomenon indicates that the charge storage process is governed by reversible redox transitions rather than purely electrostatic adsorption. These potential plateaus are associated with multi-step oxidation and reduction reactions of Co and Mn species, involving the interconversion between hydroxide and oxyhydroxide phases during the charge–discharge process. The presence of multiple plateaus suggests that several redox couples including Co^2+^/Co^3+^ and Mn^2+^/Mn^3+^ participate in the electrochemical reactions, further confirming the Faradaic nature of the electrodes. In addition, the relatively symmetric charge–discharge profiles indicate high Coulombic efficiency and good reversibility of the redox reactions. Among the three samples, CoMn-G exhibits a longer discharge time compared to Co-G and Mn-G, indicating a higher charge storage capacity, which is consistent with the CV results. This enhanced performance can be attributed to the synergistic interaction between cobalt and manganese species, which increases the number of electroactive sites and facilitates faster electron and ion transport. The *C*_F_ values are also derived from GCD measurements, as shown in Table 1. The *C*_F_ values of 430.7, 170.2, and 606.2 F/g are obtained for Co-G, Mn-G, and CoMn-G, respectively, with their corresponding specific capacities of 71.8, 28.4, and 101.0 mAh/g. Consistent with CV results, CoMn-G shows the highest capacitance, indicating superior charge storage capability. The lower performance of Mn-G can be attributed to its poor electrical conductivity, while the enhanced performance of CoMn-G arises from improved electron transport and increased electroactive sites. In addition, a noticeable IR drop can be observed in the discharge curves, which reflects the internal resistance of the electrodes.^43^ The magnitude of the IR drop follows the trend of Mn-G, Co-G and CoMn-G, indicating that Mn-G suffers from higher internal resistance and poorer electrical conductivity. In contrast, CoMn-G exhibits a much smaller IR drop, suggesting improved charge transport and reduced internal losses.

The Nyquist plots of Co-G, Mn-G, and CoMn-G electrodes are shown in Figure 4c to further evaluate the resistances in the systems. The fitted parameters obtained from the equivalent circuit are summarized in Table 1. The series resistance (*R*_s_), which represents the combined resistance of the electrolyte, intrinsic resistance of the active material, and contact resistance between the electrode and current collector,^44^ is determined to be 0.34, 0.51, and 0.47 Ω for Co-G, Mn-G and CoMn-G, respectively. The relatively higher Rs value of Mn-G indicates its poorer intrinsic conductivity, while the lower *R*_s_ values for Co-G and CoMn-G suggest improved electrical pathways. More importantly, the charge transfer resistances (*R*_ct_) of 0.90, 1.62, and 0.08 Ω are obtained for Co-G, Mn-G and CoMn-G, respectively. The charge transfer resistance corresponding to the semicircle in the high-frequency region of the Nyquist plot reflects the kinetics of Faradaic reactions at the electrode/electrolyte interface.^45^ The significantly reduced *R*_ct_ of CoMn–G indicates much faster interfacial charge transfer kinetics, which can be attributed to the synergistic interaction between Co and Mn species. The higher *R*_ct_ value for Mn-G reflects sluggish electron transfer and limited electrochemical activity. These results are consistent with the CV and GCD analyses, confirming that CoMn-G possesses superior electrochemical kinetics.

Furthermore, the electrochemical performance of the selenized samples is analyzed. The CV curves of Co–Se, Mn–Se, and CoMn-Se are shown in Figure 4d. The redox behavior of the selenized electrodes can be interpreted based on surface conversion and reconstruction reactions of metal selenium-treated products in alkaline electrolytes. During electrochemical cycling, the selenium-containing compounds undergo surface reconstruction to generate electrochemically active CoOOH and MnOOH species, which dominate the Faradaic charge storage process. The selenium species may partially dissolve or remain as an amorphous component, contributing to structural flexibility and ion transport. In the case of CoMn-Se, the coexistence of cobalt and manganese selenium-treated product phases leads to overlapping redox reactions involving both Co and Mn species. The synergistic interaction between Co and Mn facilitates faster electron transfer and enhances the utilization of electroactive sites. As a result, CoMn-Se exhibits intensified redox peaks and superior electrochemical performance compared to single-metal counterparts. Notably, all selenized electrodes exhibit significantly enhanced current densities compared to their glycerate counterparts, indicating improved electrochemical activity. The *C*_F_ values calculated from CV curves (Table 1) are 777.5, 714.5, and 927.3 F/g for Co–Se, Mn–Se, and CoMn-Se, respectively, with corresponding specific capacities of 140.4, 129.0, and 167.4 mAh/g. The relatively lower *C*_F_ value of Mn–Se compared to Co–Se can be attributed to the intrinsically poorer electrical conductivity and slower redox kinetics of Mn-based species. However, Mn–Se still shows a dramatic improvement compared to Mn-G, confirming that the selenization process effectively enhances electrical conductivity by introducing more conductive selenium-treated product phases, which facilitate faster electron transport and improve electrochemical utilization of active materials. In addition, the incorporation of selenium not only increases conductivity but also modifies the electronic structure of the metal centers, thereby promoting more reversible redox reactions. This leads to improved charge storage capability and higher utilization efficiency of electroactive sites. Among all samples, CoMn-Se exhibits the highest specific capacitance and capacity. This superior performance can be attributed to the synergistic effect between Co and Mn, where cobalt provides higher electrical conductivity and faster redox kinetics, while manganese contributes additional redox-active sites. The coexistence of these two metal species optimizes the electronic structure and facilitates charge transfer at the electrode/electrolyte interface. Furthermore, the conductive selenium-treated product framework enhances ion diffusion and reduces internal resistance, resulting in improved electrochemical performance. Overall, the enhanced capacitive behavior of CoMn-Se arises from the combined effects of improved electrical conductivity, increased density of electroactive sites, and bimetallic synergistic interactions, making it highly promising electrode material for high-performance energy storage applications.

The GCD curves of Co–Se, Mn–Se, and CoMn-Se in Figure 4e also show consistent trends as their CV results. The *C*_F_ values derived from GCD curves (Table 1) are 840.1, 715.0, and 1015.8 F/g for Co–Se, Mn–Se, and CoMn-Se, respectively, corresponding to the specific capacities of 128.3, 109.2, and 155.2 mAh/g. The prolonged discharge time of CoMn-Se further confirms its superior charge storage capability. The improvement after selenization is mainly attributed to enhanced conductivity and increased electrochemically active surface area. In addition, the IR drop observed in the GCD curves of the selenized electrodes is significantly reduced compared to their glycerate counterparts, indicating improved electrical conductivity after selenization. Among the three selenized samples, the IR drop follows the trend of Mn–Se, Co–Se and CoMn-Se. Specifically, CoMn-Se exhibits the smallest IR drop, suggesting the lowest internal resistance and most efficient charge transport behavior, while Mn–Se shows a relatively larger voltage drop, implying comparatively slower electron transfer. It should be noted that the specific capacitance obtained from CV measurements is higher than that derived from GCD measurements because the CV calculation is based on the integrated voltammetric response, whereas the GCD capacitance is determined from the discharge process under a constant current.^35,46^

The Nyquist plots of the selenized electrodes are further shown in Figure 4f, and the fitted parameters are summarized in Table 1. The *R*_s_ values of Co–Se, Mn–Se, and CoMn-Se are 0.30, 0.17, and 0.28 Ω, respectively. The relatively low *R*_s_ value of Mn–Se suggests improved intrinsic conductivity after selenization. The *R*_ct_ values are 0.05, 0.07, and 0.02 Ω for Co–Se, Mn–Se, and CoMn-Se, respectively. Notably, CoMn-Se exhibits the lowest *R*_ct_ value among all samples, indicating the fastest charge transfer kinetics. This result highlights the strong synergistic effect between Co and Mn as well as the high conductivity of selenium-treated product phases. The reduced resistance and enhanced ion diffusion collectively contribute to the superior electrochemical performance of CoMn-Se.

The rate capability and charge storage behavior of all electrodes were systematically analyzed. Figure S2­(a–c) in the SI respectively shows the CV curves of Co-G, Mn-G and CoMn-G electrodes measured at different scan rates, while Figure S2­(d–f) in the SI respectively presents their corresponding GCD curves measured at different current densities. Similarly, the CV curves at different scan rates of Co–Se, Mn–Se and CoMn-Se electrodes are respectively presented in Figure S3­(a–c) in the SI, while their corresponding GCD curves at different current densities are presented in Figure S3­(d–f) in the SI, respectively. Figure 5a shows the relationship between specific capacitances and scan rates, revealing that all samples exhibit a gradual decrease in capacitance with increasing scan rate. This phenomenon is commonly attributed to limited ion diffusion at high scan rates. Among all electrodes, CoMn-Se demonstrates the highest capacitance retention, maintaining approximately 43% of its initial capacitance at 100 mV/s. This value is significantly higher than those of Co–Se (∼38%) and CoMn-G (∼30%), indicating that the incorporation of both bimetallic components and selenium-treated product phases effectively enhances charge transport kinetics and reduces polarization under fast charging conditions. A similar trend is observed in the current density-dependent performance (Figure 5b), where CoMn-Se also exhibits superior retention compared to the other electrodes. This suggests that the CoMn-Se electrode possesses a more robust structure and improved electrical conductivity, enabling efficient electron transport and ion diffusion even at high current densities. To further elucidate the charge storage mechanism, the relationships between peak current (*i*) and scan rate (*v*) was analyzed using the power-law equation (*i* = *av* ^*b*^),^47^ and the corresponding log­(peak current) and log­(scan rate) plots for Co-G, Mn-G, CoMn-G, Co–Se, Mn–Se and CoMn-Se electrodes are respectively presented in Figure 5c–h. The extracted *b*-values provide insight into the dominant charge storage process, where a value of 0.5 indicates diffusion-controlled behavior and 1.0 corresponds to a capacitive-controlled process. As summarized in the relationships of b-value to active materials in Figure 5i, the CoMn-Se electrode exhibits *b*-values of approximately 0.45 to 0.57, suggesting that the charge storage is primarily governed by diffusion-controlled faradaic reactions with partial capacitive contribution. In contrast, Co-G shows a higher anodic *b*-value close to 1.0, indicating a more surface-controlled capacitive behavior but relatively lower overall capacitance. Notably, the selenium-treated products-based electrodes (Co–Se, Mn–Se, and CoMn-Se) generally display intermediate *b*-values, reflecting a synergistic combination of diffusion-controlled redox reactions and surface capacitive effects. This behavior can be attributed to the enhanced electrical conductivity of metal selenium-treated products and the increased availability of electroactive sites, which facilitate both rapid surface reactions and bulk ion diffusion. The enhanced electrochemical behavior of CoMn-Se can also be correlated with the electronic interaction revealed by XPS. The positive shift of the Co 2p spectrum together with the corresponding shift of Mn 2p indicates charge redistribution between the two metal species. Such electronic modulation optimizes the adsorption/desorption behavior of OH^–^ intermediates and facilitates reversible redox reactions, thereby reducing the charge-transfer resistance observed from EIS and improving both rate capability and specific capacitance. Overall, the superior rate performance of CoMn-Se can be ascribed to the combined effects of improved electrical conductivity, bimetallic synergistic interactions, and optimized charge storage kinetics, making it a promising candidate for high-performance electrochemical energy storage applications.

**Figure 5:**
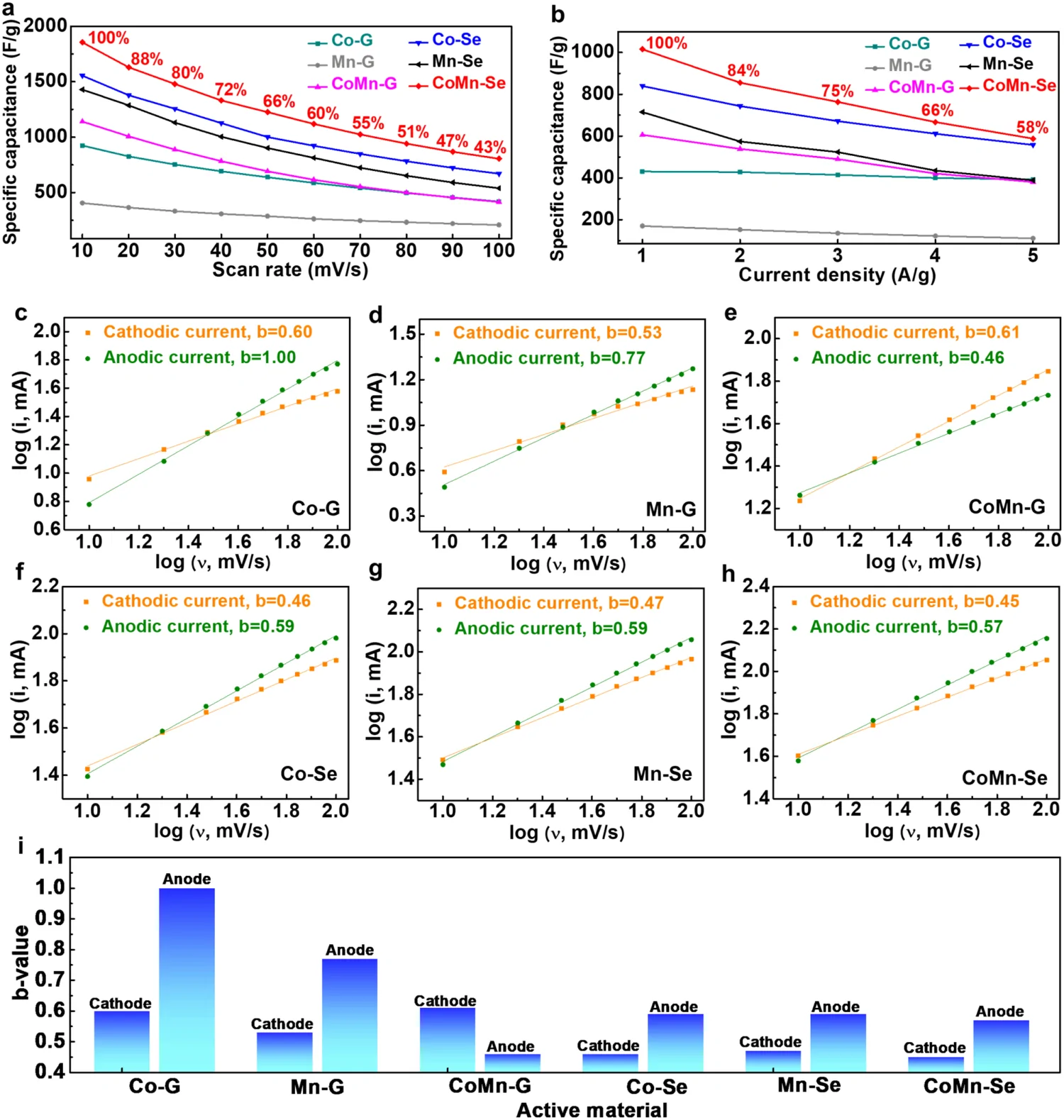
Specific capacitance versus (a) scan rate and (b) current density; (c–h) log­(current density) versus log­(scan rate); and (i) b-value as a function of active materials for Co-G, Mn-G, CoMn-G, Co–Se, Mn–Se, and CoMn-Se electrodes.

### 3.3 Electrochemical Performance Analysis of the Two-Electrode Supercapacitor

The electrochemical characteristics of the assembled two-electrode supercapacitor is systematically examined to determine a suitable operating condition and to understand its charge storage behavior. As shown in Figure 6a, CV curves were recorded at 20 mV/s under different potential windows ranging from 1.35 to 1.55 V. With increasing voltage, the enclosed area of the CV curves gradually expands, indicating enhanced charge storage capability. However, when the voltage window exceeds 1.45 V, the curve begins to show noticeable distortion along with a rapid rise in current at higher potentials, suggesting the occurrence of undesirable side reactions such as electrolyte decomposition. These effects may adversely influence long-term stability. Therefore, 1.45 V was selected as the appropriate operating voltage for this two-electrode supercapacitor. The influence of the potential window on charge/discharge behavior is further investigated by GCD measurements at 1 A/g, as illustrated in Figure 6b. At higher cutoff voltages, a more pronounced voltage drop at the beginning of discharge can be observed, indicating increased internal resistance. In contrast, the curve obtained at 1.45 V exhibits a relatively stable and symmetric profile, implying improved charge transfer efficiency and reduced energy loss during cycling. Figure 6c presents the CV curves at various scan rates within the selected voltage window of 1.45 V. The overall shapes of the curves remain consistent as the scan rate increases, indicating that the redox reactions are highly reversible. Meanwhile, the gradual increase in current response with scan rate reflects favorable kinetics and efficient ion diffusion. Although slight polarization is observed at higher scan rates, the preservation of curve features suggests that the device maintains good electrochemical stability under fast operation conditions. The corresponding GCD curves at different current densities (Figure 6d) show symmetric charge/discharge characteristics, further confirming the reversibility of the system. Even as the current density increases from 1 to 5 A/g, the profiles retain similar shapes, demonstrating that the device can operate effectively under high-rate conditions with limited performance degradation.

**Figure 6:**
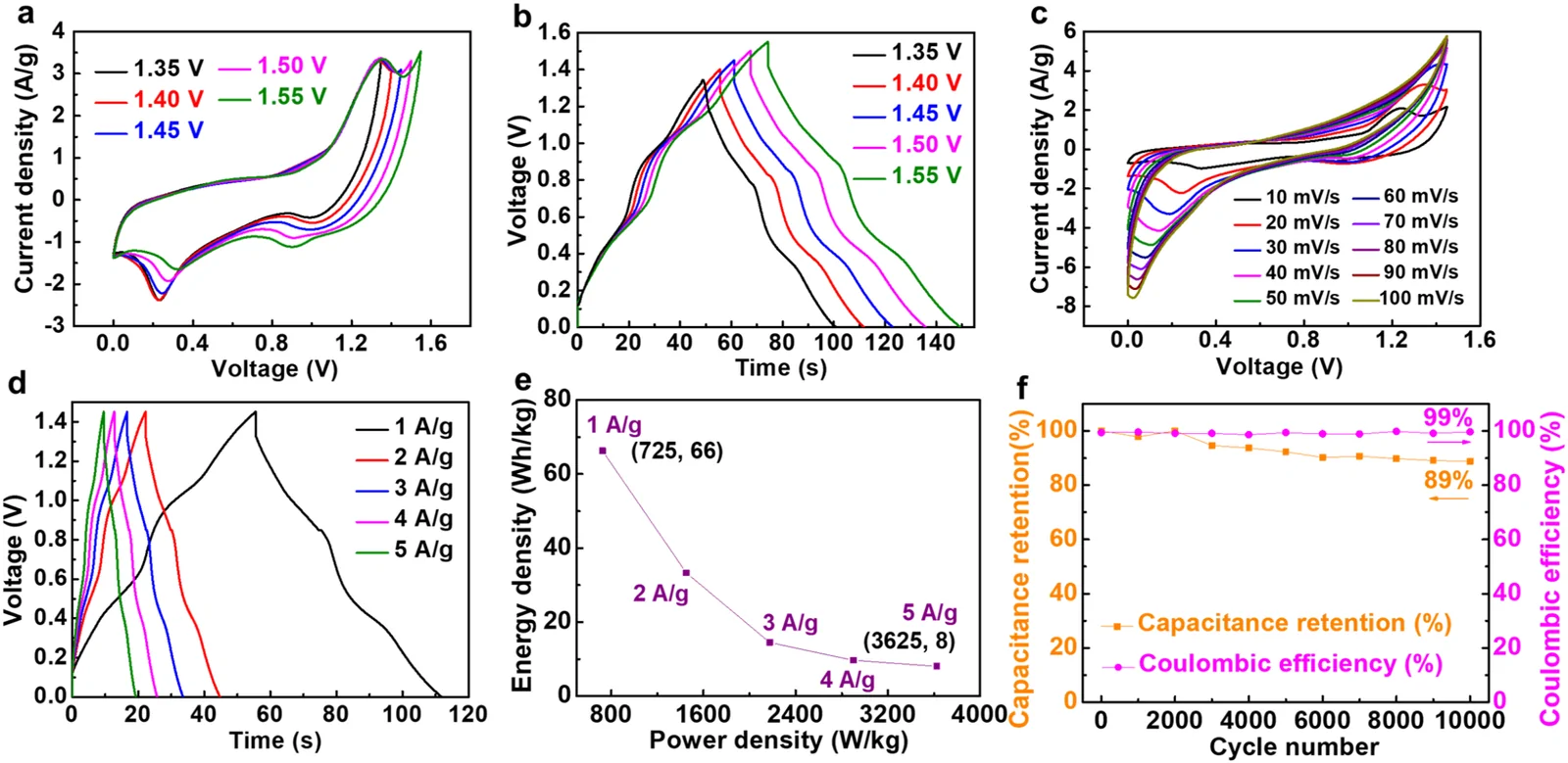
(a) CV curves at different potential windows, (b) GCD curves at different potential windows, (c) CV curves at different scan rates (1.45 V), (d) GCD curves at different current densities (1.45 V), (e) Ragone plot, and (f) cycling performance and Coulombic efficiency of the rGO//CoMn-Se asymmetric supercapacitor over 10,000 cycles at 1 A/g.

The energy and power relationship of the device is also summarized in the Ragone plot (Figure 6e). The reduced energy density is accompanied by an increase in power density. This trend reflects a typical trade-off behavior, indicating that the device is capable of delivering both high energy and high power depending on the operating conditions. At the lowest current density of 1 A/g, the device delivers a maximum energy density of 66 Wh/kg at the power density of 725 W/kg. While at the highest current density of 5 A/g, the device still achieves an energy density of 8 Wh/kg at the maximum power density of 3625 W/kg. Long-term cycling performance was eventually evaluated over 10,000 cycles at a current density of 1 A/g, as shown in Figure 6f. The capacitance retention remains at approximately 89% after 10,000 cycles, while the Coulombic efficiency is consistently maintained at around 99%. The relatively stable efficiency suggests that the charge storage process remains highly reversible, whereas the gradual capacitance decay may be associated with structural changes or minor degradation at the electrode/electrolyte interface. Overall, these results demonstrate that this two-electrode supercapacitor exhibits reliable electrochemical performance with good stability and reversibility.

The comparative results in Table 2 highlight the competitive advantages of the present CoMn–Se electrode among previously reported Co–Mn-based selenide systems. Notably, the CoMn–Se electrode delivers a high capacitance of 927.3 F/g, which is among the highest values compared to reported counterparts, indicating abundant electroactive sites and efficient redox activity. The energy density (66 Wh/kg) is competitive to reported systems, and the device also operates at a comparable power density and exhibits stable cycling performance with the capacitance retention of 89% and Coulombic efficiency of 99% after 10,000 cycles. Importantly, the use of reduced graphene oxide as the negative electrode further contributes to improved charge transport and interfacial compatibility compared to conventional active carbon-based systems. Overall, the superior capacitance, good rate performance, and stable cycling behavior suggest that synergistic effects of compositional modulation and phase reconstruction play a key role in enhancing electrochemical performance, making the proposed system competitive among current Co and Mn-based selenium-treated product electrodes.

**Table 2:** Partial Lists of Synthetic Methods and Electrochemical Performance of Supercapacitors Applying Co and Mn Based Selenium-Treated Products as Active Materials

Active material	Synthetic method	Capacity (electrode)	Negative electrode	Energy density @power density	*C*_F_ retention & Coulombic efficiency @ cycle number	refs.
Cobalt manganese selenide	In-situ deposition	519 C/g @ 1 A/g	Active carbon	35.47 Wh/kg @ 11500 W/kg	82.6% & NA @ 10,000	^48^
Co–Mn–Fe selenide	Hydrothermal process	1112.5 C/g @ 1 A/g	Active carbon	65 Wh/kg @ 802.5 W/kg	91% & 98.1% @ 10,000	^49^
Hexagonal MnCo-selenide	Hydrothermal process	270 mAh/g @ 2 A/g	Active carbon	29.39 Wh/kg @ 399.9 W/kg	97% & 98% @ 25,000	^50^
Urchin-like MnCo-selenide	Hydrothermal process	1656 F/g @ 1 A/g	Active carbon	55.1 Wh/kg @ 880 W/kg	90.2% & 97% @ 8000	^51^
Coral-like MnCo Selenide	Electro-deposition	509 C/g @ 1 A/g	Active carbon	46.2 Wh/kg @ 800 W/kg	89.2% & 100% @ 5000	^52^
MnNiCoSe spheres	Self-templating method	263.67 mAh/g @ 1 A/g	Active carbon	53.32 Wh/kg @ 1031.39 W/kg	84.28% & NA @ 10,000	^53^
MnSe_2_–CoSe_2_	Hydrothermal process	373 F/g @ 1 A/g	Active carbon	73 Wh/kg @ 750 W/kg	99% & NA @ 5000	^54^
Ni–CoSe_2_	Hydrothermal process	1022 F/g @ 1 A/g	Active carbon	37 Wh/kg @ 3809 W/kg	98.58% & NA @ 10,000	^55^
CoMn-Se	Solvothermal process	927.3 F/g (167.4 mAh/g) @ 10 mV/s	Reduced graphene oxide	66 Wh/kg @ 725 W/kg	89% & 99% @ 10,000	this work
		1015.8 F/g (155.2 mAh/g) @ 1 A/g				

## 4 Conclusions

This study presents a strategy for engineering cobalt- and manganese-based electrode materials via compositional regulation and selenization for supercapacitor applications. The incorporation of Mn suppresses the crystallization of Mn oxide phases, resulting in a homogeneous amorphous precursor, while subsequent selenization induces phase reconstruction into CoSeO_4_-dominant structures with Mn­(SeO_3_)_2_ and a porous architecture. Such structural evolution enhances electrolyte accessibility and interfacial redox activity. Accordingly, the CoMn-Se electrode delivers a high specific capacitance of 927.3 F/g with a low charge transfer resistance of 0.02 Ω. The assembled two-electrode supercapacitor device operates at 1.45 V, achieving a maximum energy density of 66 Wh/kg at 725 W/kg, and maintaining the capacitance retention of 89% and Coulombic efficiency of 99% after 10,000 cycles. More importantly, the present glycerolate-derived interfacial engineering strategy is not limited to the Co–Mn system but can be readily extended to other multimetallic transition-metal compounds for advanced energy-storage applications. Future studies focusing on high-mass-loading electrodes, flexible configurations, and large-scale device fabrication are expected to further promote the practical implementation of this material design strategy.

## Supplementary Material



## Notes

High-resolution XPS spectra of Co–Se, Mn–Se, and CoMn-Se, as well as CV and GCD curves of Co-G, Mn-G, CoMn-G, Co–Se, Mn–Se, and CoMn-Se electrodes (PDF)
