# Supplementary material for: Interfacial Coupling and Reconstruction in Glycerolate-Derived Co–Mn Selenium-Treated Products for High-Performance Supercapacitors
Source: ACS Omega. 2026 Sep 2;11(36):54704–18. doi: 10.1021/acsomega.6c07974 (PMC13584563; doi:10.1021/acsomega.6c07974)
Supplement: Supplementary file 1 [file ao6c07974_si_001.pdf]

Supporting Information

Interfacial Coupling and Reconstruction in Glycerolate-Derived Co–Mn Selenium-treated Products for High-Performance Supercapacitors

Tsai-Mu Cheng[a,b,c*], Yu-Yan Ciou[d], Yan-Yu Liu[d], Chutima Kongvarhodom[e], Sibidou Yougbaré[f], Muhammad Saukani[g], Hung-Ming Chen[h], and Lu-Yin Lin[d*]

[a]Graduate Institute for Translational Medicine, College of Medical Science and Technology, Taipei Medical University, Taipei 11031, Taiwan

[b]Taipei Heart Institute, Taipei Medical University, Taipei 11031, Taiwan

[c]Cardiovascular Research Center, Taipei Medical University Hospital, Taipei 11031, Taiwan

[d]Department of Chemical Engineering and Biotechnology, National Taipei University of Technology, Taipei, Taiwan

[e]Department of Chemical Engineering, King Mongkut's University of Technology Thonburi, 126 Pracha-u-thit, Toong-kru, Bangkok 10140, Thailand

[f]Institut de Recherche en Sciences de la Santé (IRSS-DRCO)/Nanoro, 03 B.P 7192, Ouagadougou 03, Burkina Faso

[g]Department of Mechanical Engineering, Faculty of Engineering, Universitas Islam Kalimantan MAB, Jl. Adhyaksa No. 2, Banjar-masin 70124, Kalimantan Selatan, Indonesia

[h]Gingen technology Co., LTD., Rm. 7, 10F., No.189, Sec. 2, Keelung Rd., Xinyi Dist., Taipei 11054, Taiwan

*Corresponding author (T.M. Cheng): E-mail: tmcheng@tmu.edu.tw

*Corresponding author (L.Y. Lin): E-mail: lylin@ntut.edu.tw

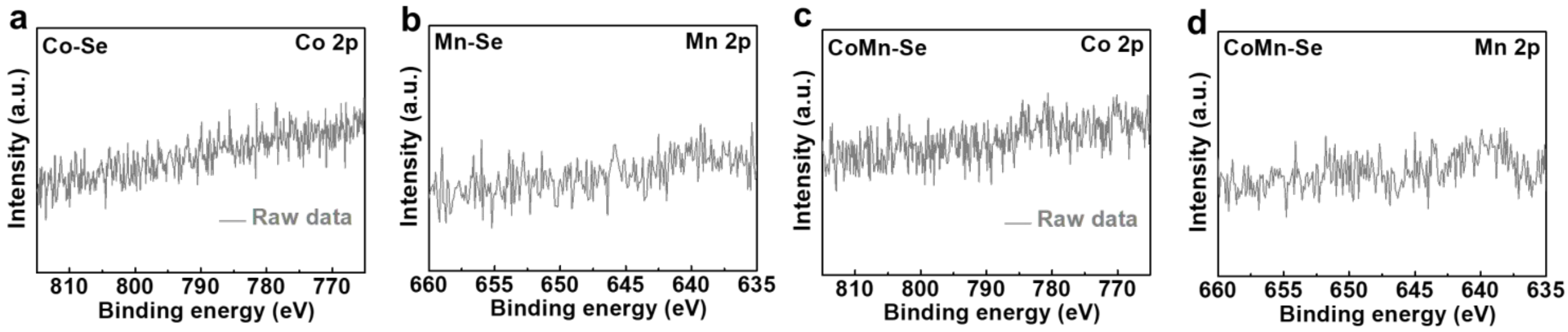

Figure S1 (a) Co 2p spectra of Co-Se, (b) Mn 2p spectra of Mn-Se, (c) Co 2p and (d) Mn 2p spectra of CoMn-Se.

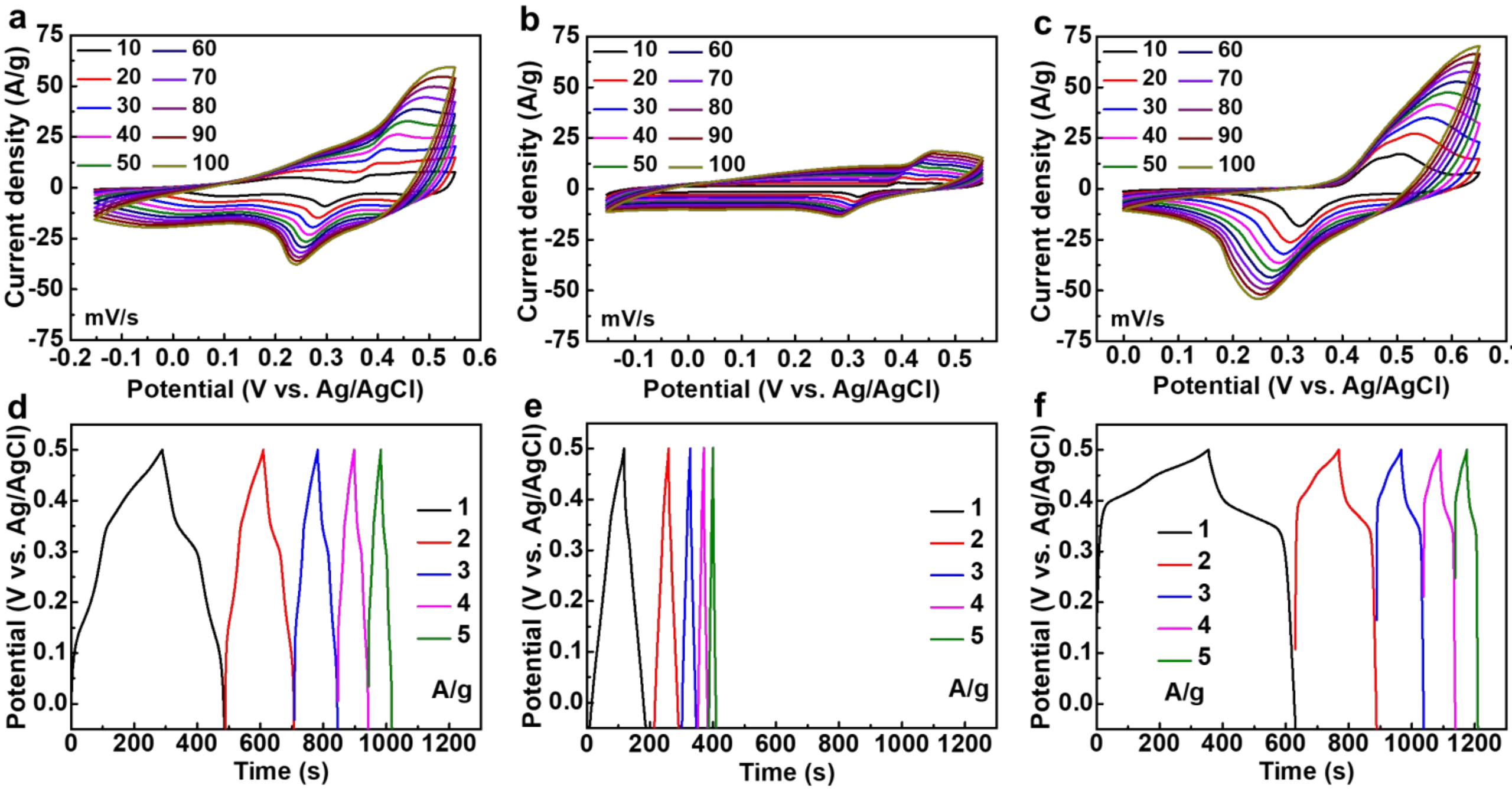

Figure S2 CV curves of (a) Co-G, (b) Mn-G and (c) CoMn-G electrodes; GCD curves of (d) Co-G, (e) Mn-G and (f) CoMn-G electrodes.

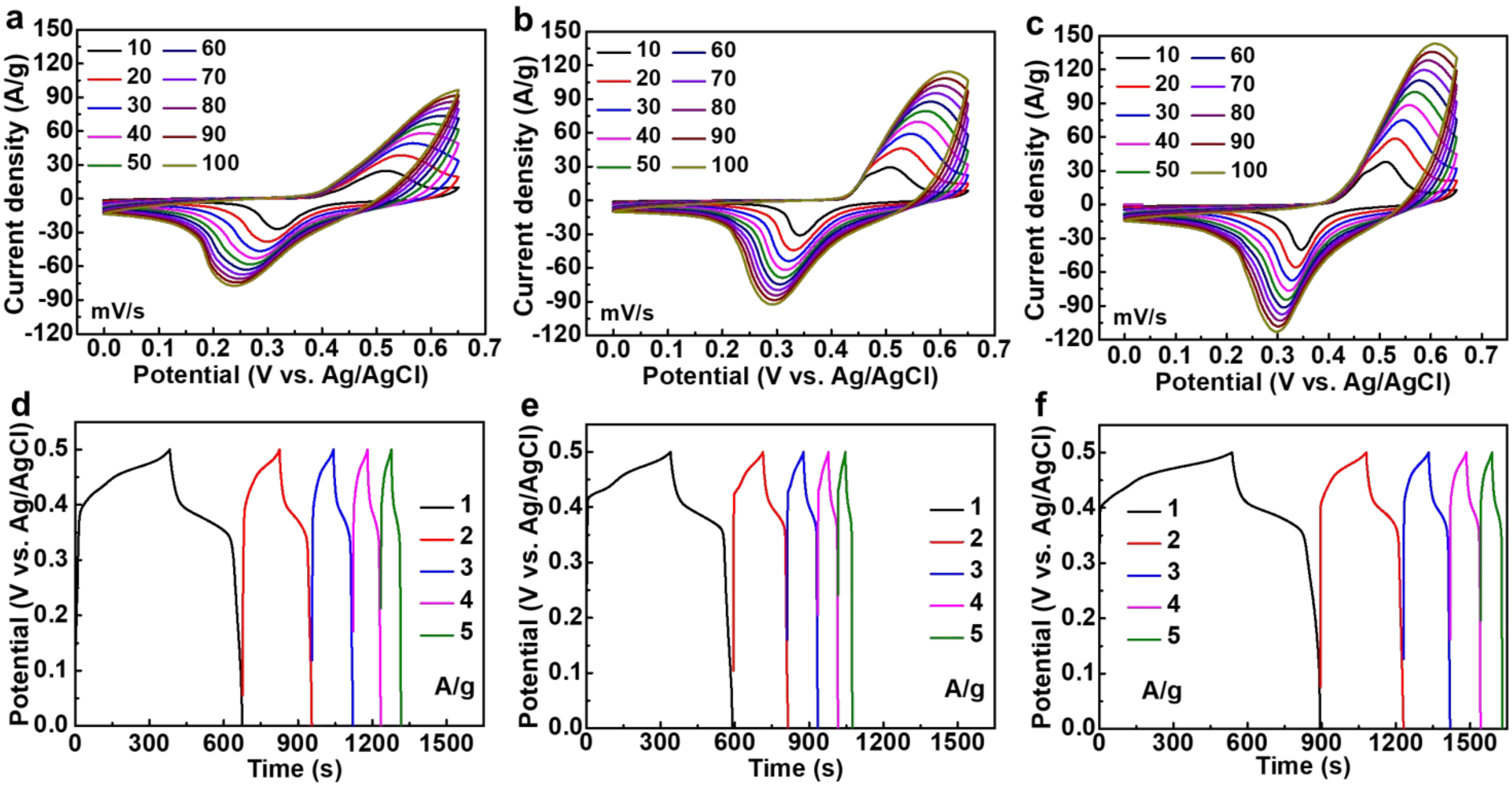

Figure S3 CV curves of (a) Co-Se, (b) Mn-Se and (c) CoMn-Se electrodes; GCD curves of (d) Co-Se, (e) Mn-Se and (f) CoMn-Se electrodes.
